# Ground surface settlement analysis of shield tunneling under spatial variability of multiple geotechnical parameters

**DOI:** 10.1016/j.heliyon.2019.e02495

**Published:** 2019-09-21

**Authors:** Baolin Hu, Changhong Wang

**Affiliations:** Department of Civil Engineering, Shanghai University, 333 Nanchen Road, Shanghai, 200444, China

**Keywords:** Civil engineering, Structural engineering, Geotechnical parameter, Ground surface settlement, Reliability index, Spatial variability, Stochastic simulation, Risk analysis, Soil engineering, Construction engineering, Foundation engineering, Computer-aided engineering

## Abstract

This paper presents an efficient method of shield tunneling reliability analysis using spatial random fields. We introduced two stochastic methods into numerical simulation. The first one computes the maximal ground surface settlement using classical statistics, in which the response surface method is utilized to calculate the failure probability by first-order second moment. Cohesion, internal friction angle, Young's modulus and mechanical model factor are considered as random variables. The second method is the spatial random fields of aforementioned three key geotechnical parameters. Using these two methods, similar multiple soil layers are converted into a stationary random field by local regression as the first step, and then the process is followed by the spatially conditional discretization of multivariate. Failure probability of maximal ground surface settlement is calculated by a subset Monte-Carlo Algorithm. This approach is applied into the four-overlapping shield tunnels of the 5^th^ and 6^th^ metro lines intersecting at Huanhu W Rd station, Tianjin China. The failure analysis results indicated that classical statistics of geotechnical parameters showing higher variability than spatial random fields, which substantially support the complex shield tunneling project.

## 1 Introduction

In recent years, many shield tunneling projects have been built in the congested urban areas, which often involve excavation of multiple proximal tunnels. Engineers are required to control the ground surface settlement caused by tunneling process, which is the prerequisite for avoiding excavation collapses. Suwansawat and Einstein (2006) used artificial neural networks to predict the maximal ground surface settlement, which enables us to non-linearly map the input factors into multi-target recognition. Moreover, Suwansawat and Einstein (2007) described the settlement troughs over side-by-side or stacked twin tunnels using superposition technique. However, experimental results should be depended on more validations of soil mechanics.

Stability analysis of ground surface settlement due to shield tunneling process is commonly performed by deterministic approaches (Loganathan and Poulos, 1998; Pinto and Whittle, 2013; Ibrahim et al., 2015), and a probability-based unneling analysis is reasonable since it enables us to consider intrinsic uncertainty of geotechnical parameters (Zhang, Chen and Huang, 2005; Mollon et al., 2009). Furthermore, spatial variability is pertinent to the geomaterial due to aleatory and structural uncertainties that coexist innately (Wang and Zhu, 2016; Wang et al., 2016). Therefore, probability-based researches are mainly divided into three categories, which are uncertainty evaluation of geotechnical parameters, bias estimation of mechanical models, and reliability analysis of soil mechanics.

Studies show that deterministic analysis of geotechnical parameters might lead to overestimating safety of shield tunneling process. Thus, the methods used to reduce the uncertainty, and thereby minimize unforeseeable risks, are classified into two sub-categories. The first one adopts an empirical relation or statistical correlation between the geotechnical parameters, which include the undrained shear strength and Young's modulus, and the in-situ or laboratory test results. This is supported by various researchers in the literature (Ching and Chen, 2007; Cao and Wang, 2014; Ching et al., 2016; Phoon et al., 2016). The next step is implemented by field measurements, which are used to reduce the uncertainty of geotechnical parameters by back analysis (Finno and Calvello, 2005; Hashash et al., 2010; Juang et al., 2012).

Moreover, bias of different constitutive equations varies dramatically because each model concentrates on the unique mechanical behaviors. Sakurai (1997) brought field measurements into modeling mechanical properties of soils and rocks. The author emphasized that the mechanical model should be calibrated by the back analysis. For instance, elastic-plastic model bias of Mohr-Coulomb failure criterion was calibrated by his work. Furthermore, Sakurai et al. (2003) identified the multi-source data and back analysis schema of tunneling process, and thus, linear and nonlinear soil behaviors are simulated by several mechanical models. The best approach is performed consistently in terms of predictions and measurements. Therefore, reliability analysis would be considered by the above two basic approaches. For example, Mollon et al. (2009) analyzed the failure probability of a pseudo shield tunneling case using First-Order Second Moment (FOSM) in a homogeneous soil layer, and concluded that cohesion, internal friction angle, and Young's modulus of surrounding soil mass have significant impacts on ground surface settlements. Papaioannou and Straub (2012) updated the displacement reliability of a foundation excavation using spatial variability of Young's modulus in a uniform soil layer, and it is highlighted by a Markov Chain Monte-Carlo algorithm to accelerate stochastic simulation (Ching and Chen, 2007). Qi and Zhou (2017) proposed an efficient Bayesian method for geotechnical parameters using displacements of the diaphragm. Hence, model factor and measurement errors were assumed so that the predictions made using the updated geotechnical parameters are agreed fairly well with the displacements.

Spatial random fields is advantageous because: (1) it transforms non-stationary soil layers into Gaussian random fields using local regression, (2) it provides a conditional discretization of multivariate utilizing SGS; and (3) it efficiently calculates the failure probability using Monte-Carlo acceleration algorithm.

The 5^th^ and 6^th^ metro lines of four-overlapping shield tunnels are demonstrated in Tianjin, China. Spatial random fields are employed to depict the uncertainty of key geotechnical parameters. For comparison, spatial variability is simplified into classical statistics. Response Surface Method (RSM) and Monte-Carlo (MC) simulation would be used to calculate the failure probability. On the other hand, SGS and Subset Monte-Carlo (SMC) simulation of spatial random fields are utilized for more efficient calculation.

## 2 Background

Limit state function G of maximal ground surface settlement induced by shield tunneling process is,

(1) $$G=v_{\max}-v\text{,}$$

**Fig. 1:**
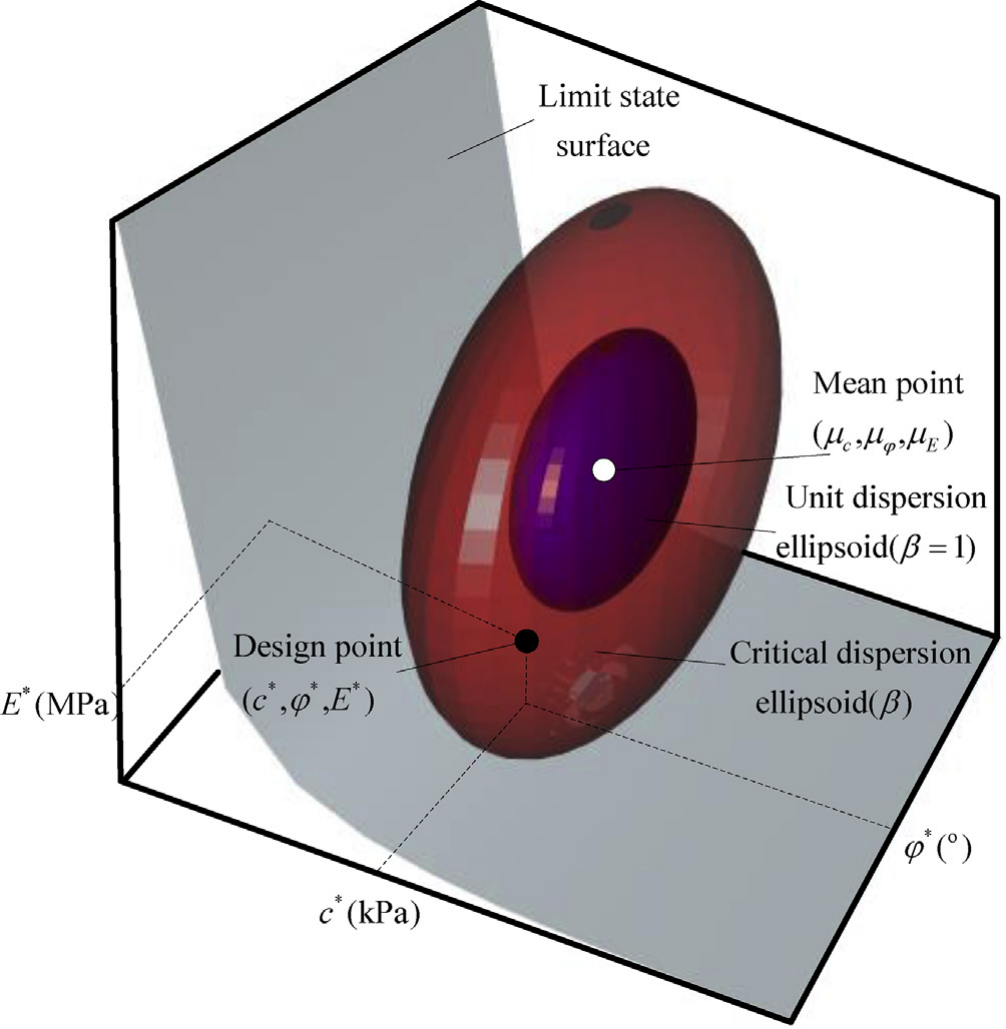
Reliability index is a function of three key geotechnical parameters, cohesion c(kPa), internal friction angle φ (^0^) and Young's modulus E(MPa).

(2) v=χ⋅δ(θ),

where, $v_{\max}$ denotes the specialized threshold. v is the theoretically maximal settlement of the ground surface; model factor χ is the deviation ratio of actual settlement divided by the prediction; θ denotes the random variables (*e.g*., cohesion c, internal friction angle φ and Young's modulus E); δ(θ) is the maximal settlement of ground surface calculated by forward analysis (*e.g*., empirical equation, finite element method, and finite difference method), and the failure probability index β could be represented by $P_{f}[G\leq \text{0]}$ as shown in Eq. (3). It is pictorially presented by Fig. 1 (Low and Tang, 2007).

(3) $$P_{f}=\text{ ​}\Phi \left[-\beta \right]\text{,}$$

where, operator Φ[⋅]denotes the cumulative Gaussian distribution.

### 2.1 Response surface method

According to classical statistics, failure probability with independent Gaussian variables is given as:

(4) $$\beta =\frac{G{|}_{\theta }+\sum_{i=1}^{n}\frac{\partial G}{\partial \theta_{i}}{|}_{\theta }\cdot \left(\mu_{\theta_{i}}-\theta_{i}\right)}{\sqrt{\sum_{i=1}^{n}{\left[\frac{\partial G}{\partial \theta_{i}}{|}_{\theta }\cdot \sigma_{\theta_{i}}\right]}^{2}}}\text{,}$$

where, θ represents n design points (*e.g*., $\theta_{1},\theta_{2},\theta_{3}=c$, φ, E and $\theta_{4}=\chi$), $\mu_{\theta }$ denotes the mean values, and $\sigma_{\theta }$ is the Standard Deviation (SD) vector. It is worth stressing that non-Gaussian and correlated multivariate could be transformed into Eq. (4) by necessary probabilistic derivation. RSM is intended to calculate the failure probability and the design points by numerical simulation, which commonly uses a quadratic polynomial without cross terms (Tandjiria et al., 2000; Li et al., 2015), and it has been applied into various mechanical problems such as slope stability and tunneling risk analysis as shown in Eq. (5).

(5) $$\delta \left(\theta \right)=g_{0}+\sum_{i=1}^{3}a_{i}\theta_{i}+\sum_{i=1}^{3}b_{i}\theta_{i}^{2}\text{,}$$

where, $\theta_{1},\theta_{2},\theta_{3}$ denote the random variables (*e.g*., c, φ and E); $g_{0}$, $a_{1},a_{2},a_{3}$ and $b_{1},b_{2},b_{3}$ are the constant coefficients of quadratic polynomial. Note that a second-order item without cross terms $\theta_{i}\theta_{j}\left(i\neq j\right)$ is adopted to calculate the maximal settlement of the ground surface. In this case, although a higher-order polynomial could also be applied, the effort required to obtain the unknown coefficients would increase significantly.

### 2.2 Algorithm schema

**Fig. 2:**
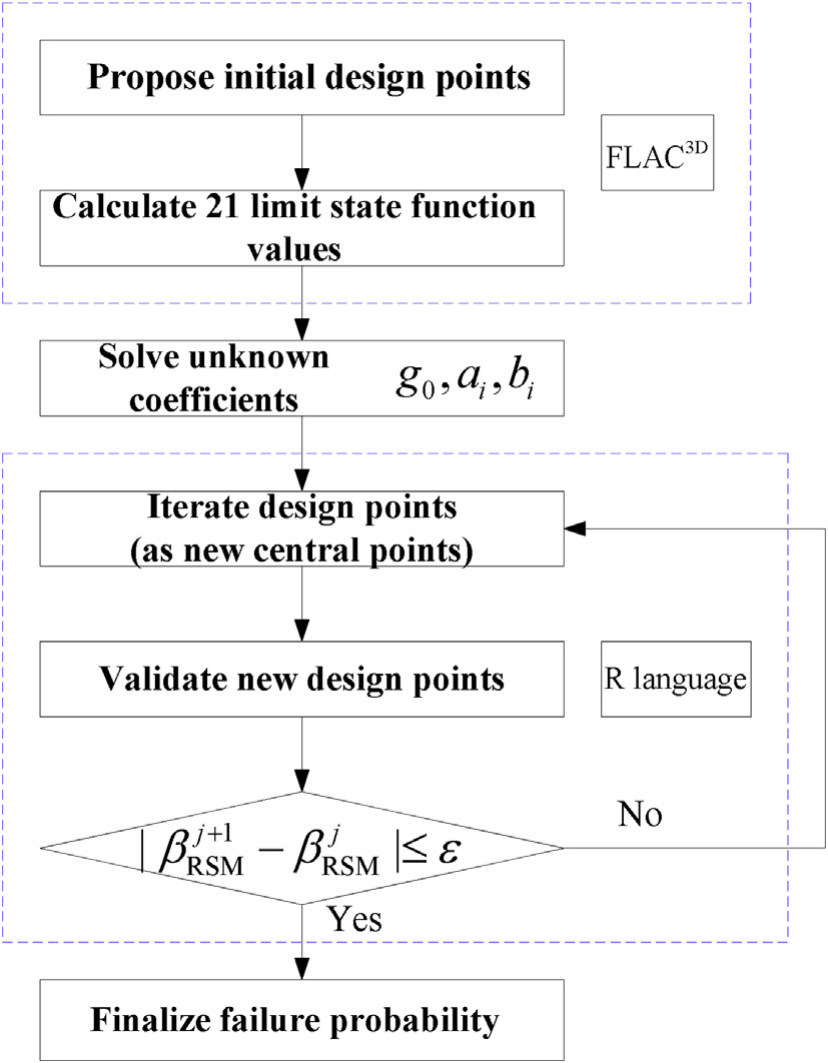
Reliability analysis using RSM of classical statistics.

Unknown coefficients $g_{0}$, $a_{1},a_{2},a_{3}$ and $b_{1},b_{2},b_{3}$ of the quadratic polynomial are calculated by RSM as shown in Fig. 2. Algorithm is composed of the following four steps:

(1) Selecting the mean values $\mu_{c}$, $\mu_{\varphi }$, $\mu_{E}$, and 6 samples $\mu_{c}\pm 2\sigma_{c}$, $\mu_{E}\pm 2\sigma_{E}$ and $\mu_{\varphi }\pm 2\sigma_{\varphi }$, as well as multiplying χ (*i.e.*, with mean value $\mu_{\chi }$ and $\mu_{\chi }\pm 2\sigma_{\chi }$) to calculate the limit state function G as shown in Eq. (1).
(2) Building 21 linear equations $G_{i}$, $c_{i}$, $\varphi_{i}$, $E_{i}$ and $\chi_{i}$, i=1,...,21, which helps to solve out the unknown coefficients $g_{0}$,$a_{1},a_{2},a_{3}$ and $b_{1},b_{2},b_{3}$ using the least square method.
(3) Denoting initial design points $\theta^{\left(0\right)}$ as $\mu_{\theta }$, obtaining design points $\theta^{\left(1\right)}$ and calculating the failure probability index $\beta^{1}$, and replacing the central points $\mu_{\theta }^{1}$ with the design points $\theta^{\left(1\right)}$ as shown in Eq. (4).
(4) And finally, repeating step 3 until convergence is achieved by the value $\left|\beta^{j}-\beta^{j-1}\right|\leq \varepsilon$ with the design points $\theta^{\left(j\right)}$.

## 3 Theory

Spatial random fields emphasize the auto-correlation and cross-correlation of geotechnical parameters. It could support more accurate reliability analysis using stationary field, spatially conditional discretization and stochastic simulation.

### 3.1 Stationary process

Note that numerical analysis of soil mechanics requires customarily multiple geotechnical parameters, such as cohesion c, internal friction angle φ and Young's modulus E, and the number of input variables might increase if several soil layers are involved, in which case each soil layer possesses a set of geotechnical parameters. Hence, realization of spatial random fields always involves the high-dimensional sampling schema. Therefore, local regression is a common way to obtain a Gaussian field (Wang et al., 2017). Accordingly, a geotechnical parameter could be expressed as,

(6) Z(x)=μ(x)+ε(x),

where, μ(x) denotes the stepped-drift of borehole data, and ε(x) represents the zero-mean error subjecting to Gaussian distribution. The variance could adopt the thickness-weighted value of the multiple soil layers as shown in Eq. (7).

(7) $$\sigma^{2}=\frac{\sum_{i=1}^{L}\sigma^{2}\left(x_{i}\right)\cdot H_{i}}{\sum_{i=1}^{L}H_{i}}\text{ ​,}$$

where, L is the number of soil layers. $H_{i}$ denotes the corresponding thickness of the $i^{th}$ soil layer, i=1,...,L, and $\sigma^{2}$ represents the weighted variance of a Gaussian field. The spatial variability of a geotechnical parameter could be depicted by experimental variogram (Matheron and Armstrong, 1963), which is calculated by residual data $\varepsilon \left(x_{i}\right),\text{ ​}i=1,...,n$ as shown below,

(8) $$\gamma^{*}\left(h\right)=\frac{1}{2N_{h}}\sum_{i=1}^{N_{h}}{\left[\varepsilon \left(x_{i}+h\right)-\varepsilon \left(x_{i}\right)\right]}^{2}\text{,}$$

where, h is the lag distance. $N_{h}$ denotes the paired data number between $\left(x_{i}+h,x_{i}\right)$ to calculate experimental variogram.

There are several well-known theoretical variogram, exponential, Gaussian, and Spherical models are among the common ones. For instance, the exponential model is one of them, which is adaptive to the scenario of longer range, the Gaussian model could depict the reverse shape of a variogram, whereas the Spherical model (Vanmarke, 1977) has the advantages of conciseness and robustness; hence it is applied widely into practice as indicated in Eq. (9).

(9) $$\gamma \left(h\right)=\text{ ​ ​ ​}\{\begin{array}{l} C_{0}+C_{1}\left[{1.5\left(h/a\right)-0.5\left(h/a\right)}^{3}\right]\text{ ​ ​ ​0}\leq h\leq a\\ C_{0}+C_{1},\text{ ​ ​ ​ ​ ​ ​ ​ ​ ​ ​ ​ ​ ​ ​ ​ ​ ​ ​ ​ ​}h>a \end{array}\text{,}$$

where, $C_{0}$ defines the nugget, $C_{0}+C_{1}$ equals the variance $\sigma^{2}$ , and a denotes the range. Fitting package ‘*gstat*’ of R language (Pebesma, 2004) would solve out the unknown coefficients from experimental variogram. Furthermore, the three-dimensional heterogeneity of a geotechnical parameter would be taken into account by the weighted lag distance h (Matheron and Armstrong, 1963),

(10) $$h=\sqrt{{\left(\frac{h_{1}}{\eta_{1}}\right)}^{2}+{\left(\frac{h_{2}}{\eta_{2}}\right)}^{2}+{\left(\frac{h_{2}}{\eta_{3}}\right)}^{2}}\text{,}$$

where, $\eta_{1}$ equals to 1.0 constantly (*i.e*., on the horizontal coordinate X1), $\eta_{2},\text{ ​}\eta_{3}$ are the ratios of the second (*i.e*., on the horizontal coordinate X2), third (*i.e*., on the vertical coordinate X3) ranges $a_{2},a_{3}$ divided by the first range $a_{1}$_._

### 3.2 Spatially conditional discretization

The difference between random variables and random fields is that the latter one needs spatial discretization for a stochastic analysis. To this end, several well-known decomposition methods are implemented. Some of the decomposition methods have been successfully achieving the unconditional discretization include Cholesky decomposition (Dariusz and Marek, 2004), turning band method (Mantoglou and Wilson, 1982), Local average method (Zhu et al., 2015), Karhunen-Loeve decomposition method (Huang et al., 2001) and random harmonic function (Liang et al., 2013). However, the algorithms used in these methods do not handle multivariate conditional discretization of spatial random fields.

Let us generalize the SGS for multivariate, in which the bivariate $\varepsilon_{1}\left(x\right)$, $\varepsilon_{2}\left(x\right)$ are demonstrated (Pebesma, 2004). Known quantity in this case includes zero-mean values, variance $\sigma_{\varepsilon_{1}}^{2}$ and $\sigma_{\varepsilon_{2}}^{2}$, variogram $\gamma_{11}\left(h\right)$ and $\gamma_{22}\left(h\right)$, as well as cross-variogram $\gamma_{12}\left(h\right)=\gamma_{21}\left(h\right)$. And the prediction of $\varepsilon_{2}\left(x_{0}\right)$ is calculated in terms of residual data $\varepsilon_{1}$ and $\varepsilon_{2}$ as shown below,

(11) $$\gamma_{12}\left(h\right)=\sigma_{\varepsilon_{1}}\sigma_{\varepsilon_{2}}-C_{12}=\frac{1}{2}E\left\{\left[\varepsilon_{1}\left(x+h\right)-\varepsilon_{1}\left(x\right)\right]\left[\varepsilon_{2}\left(x+h\right)-\varepsilon_{2}\left(x\right)\right]\right\}\text{,}$$

(12) $$\varepsilon_{2}^{*}\left(x_{0}\right)=\sum_{i=1}^{n_{1}}\lambda_{1i}\varepsilon_{1}\left(x_{i}\right)+\sum_{j=1}^{n_{2}}\lambda_{2j}\varepsilon_{2}\left(x_{j}\right),\text{ ​ ​}\sum_{i=1}^{n_{1}}\lambda_{1i}=0,\sum_{j=1}^{n_{2}}\lambda_{2j}=1\text{,}$$

(13) $$\sigma_{2}^{2}\left(x_{0}\right)=\sum_{i=1}^{n_{1}}\lambda_{1i}r_{12}\left(x_{i}-x_{0}\right)+\sum_{j=1}^{n_{2}}\lambda_{2j}r_{22}\left(x_{j}-x_{0}\right)+\xi_{2}\text{,}$$

where, $\sigma_{\varepsilon_{1}}$, $\sigma_{\varepsilon_{2}}$ is the SD of variable $\varepsilon_{1}\left(x\right)$, and $\varepsilon_{2}\left(x\right)$, respectively. $C_{12}$ is the covariance, which could be derived by correlation coefficient $\rho_{12}$ multiplying $\sigma_{\varepsilon_{1}}\sigma_{\varepsilon_{2}}$. $n_{1}$ and $n_{2}$ are the adjacent conditional data. We may practically confine $n_{1},n_{2}=20$. Interpolation coefficients $\lambda_{1i},i=1,...,n_{1}$ and $\lambda_{2j},j=1,...,n_{2}$, Lagrangian coefficients $\xi_{1},\xi_{2}$ are determined by the linear Co-Kriging equations (Eldeiry and Garcia, 2010).

(14) $$\begin{array}{l} \sum_{i=1}^{n_{1}}\lambda_{1i}\gamma_{11}\left(x_{1i}-x_{I}\right)+\sum_{j=1}^{n_{2}}\lambda_{2i}\gamma_{21}\left(x_{2j}-x_{I}\right)+\xi_{1}=\gamma_{11}\left(x_{0}-x_{I}\right),I=1,2,...,n_{1}\\ \sum_{i=1}^{n_{1}}\lambda_{1i}\gamma_{12}\left(x_{1i}-x_{J}\right)+\sum_{j=1}^{n_{2}}\lambda_{2i}\gamma_{22}\left(x_{2j}-x_{J}\right)+\xi_{2}=\gamma_{22}\left(x_{0}-x_{J}\right),J=1,2,...,n_{2} \end{array}$$

**Fig. 3:**
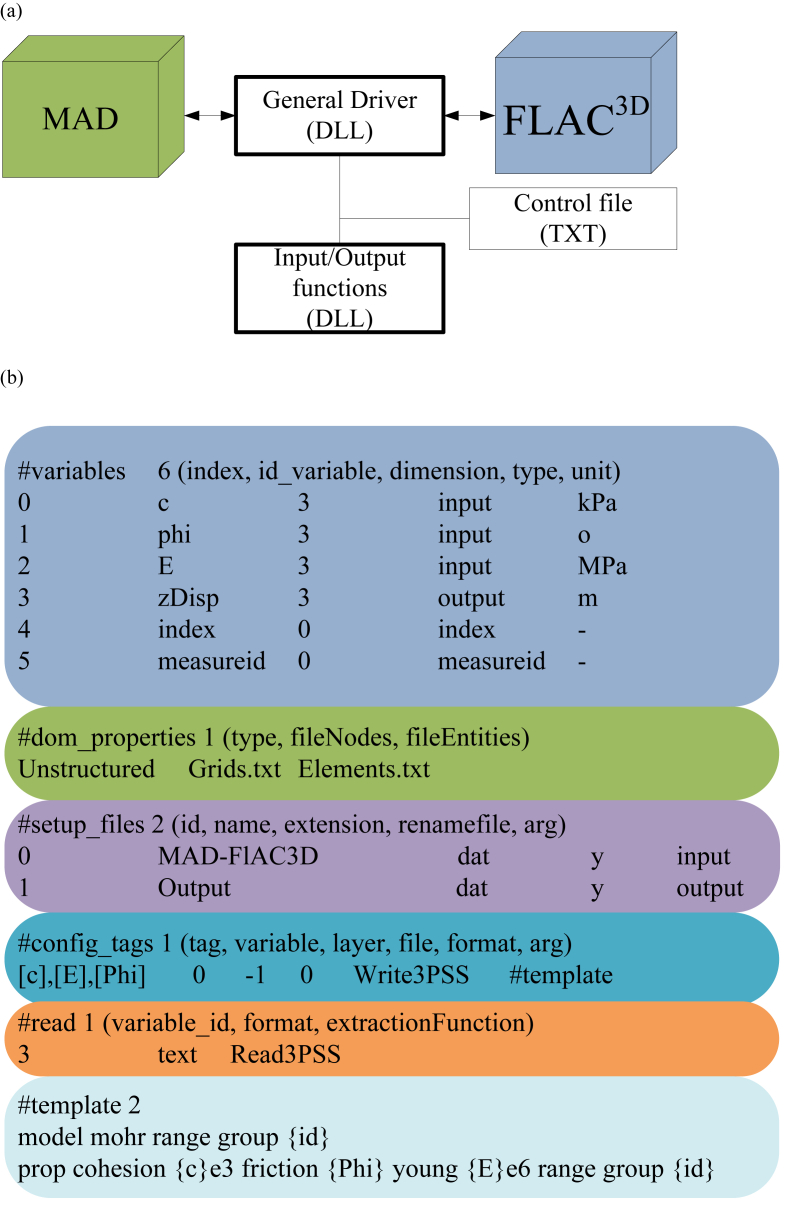
Master-slave framework of connecting open source MAD and commercial software FLAC^3D^. (a) Controlling schema of the general master driver; and (b) Configuration template of FLAC^3D^.

Specific prediction $\varepsilon_{2}\left(x_{0}\right)$ would be assumed to comply with Gaussian distribution$N\left[\varepsilon_{2}^{*}\left(x_{0}\right),\sigma_{2}^{2}\left(x_{0}\right)\right]$. And the same conclusion is established to the prediction $\varepsilon_{1}\left(x_{0}\right)$ at point $x_{\text{0}}$. In order to obtain a conditional discretization of the Gaussian fields, SGS denotes the previous samples as conditional data for a new point simulation. On the other hand, multivariate discretization of the spatial random fields is handled by the Method of Anchored Distribution (MAD) (Rubin et al., 2010; Osorio-Murillo et al., 2015), and by communicating with the software FLAC^3D^. A general driver is designed innovatively here to link MAD with FLAC^3D^ according to the master-slave schema as shown in Fig. 3(a). The configuration of FLAC^3D^ relies on a control file, and several input and output functions, and the major content of the control file is listed in Fig. 3(b). Tag *#variable* informs MAD to link input or output functions, *#dom_properties* describes the spatial domain, *#setup_files* specifies the input and output files of FLAC^3D^, and the *#config_tags* uses the input function *Write3PSS* to update the discrete values of spatial random fields according to the definition of *#template*, such that FLAC^3D^ could execute numerical analysis. Finally, tag *#read* enables output function *Read3PSS* to read the analytic predictions of the tunneling measurements. As such, the algorithm of SGS is depicted as follows:

(1) Getting the residual error ε by subtracting stepped-drift μ from the borehole data z.
(2) Defining a random path at the midpoints of Kstochastic elements, if and only if each element could be traversed once a time, conditional data are consisted of the original residual data $\varepsilon_{i},i=1,2,...,n$ and the previous realizations $\varepsilon \left(x_{1}\right),...,\varepsilon \left(x_{k-1}\right)$, k=1,2,...,K.
(3) Predicting the mean value $\varepsilon_{1}^{*}\left(x_{k}\right)$, $\varepsilon_{2}^{*}\left(x_{k}\right)$ and variances $\sigma_{1}^{2}\left(x_{k}\right)$, and $\sigma_{2}^{2}\left(x_{k}\right)$ at the midpoint $x_{k}$ given the conditional data $\varepsilon_{i},i=1,2,...,n$, variogram $r_{11}\left(h\right)$, $r_{22}\left(h\right)$ and cross-variogram $r_{12}\left(h\right)$.
(4) Building two Gaussian distribution $N\left[\varepsilon_{1}^{*}\left(x_{k}\right),\sigma_{1}^{2}\left(x_{k}\right)\right]$ and $N\left[\varepsilon_{2}^{*}\left(x_{k}\right),\sigma_{2}^{2}\left(x_{k}\right)\right]$ at the midpoint $x_{k}$, which enable random sampling to get one value, and the computed values are used as conditional data for the subsequent midpoints.
(5) Moving to the next midpoint $x_{k+1}$ and repeating the above steps 3–4 until the overall Kstochastic elements are iterated.
(6) Finishing one full cycle of conditional realization of the spatial random fields.

### 3.3 Classical Monte-Carlo Simulation

**Fig. 4:**
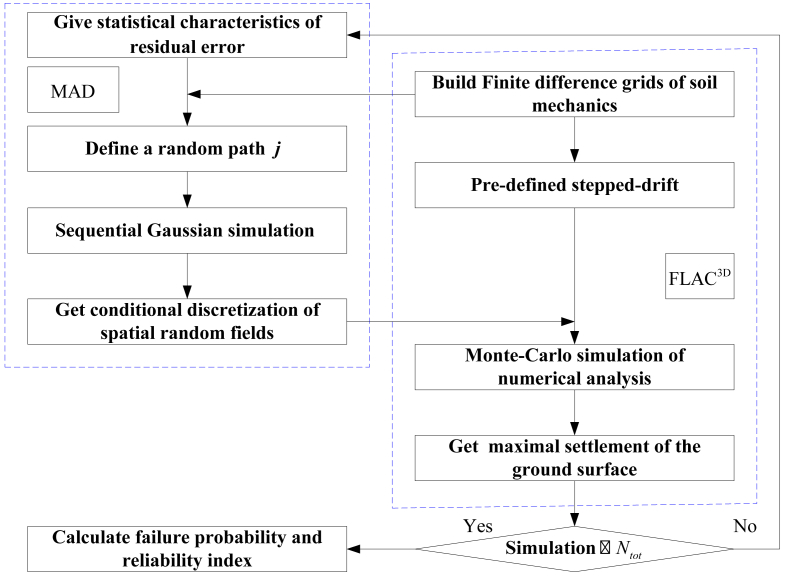
Reliability analysis using MC simulation of spatial random fields.

As mentioned in the preceding section, discretization and reliability analysis of spatial random fields could not employ RSM because it is only effective for random variables. Hence, MC simulation of spatial random fields is run directly on FLAC^3D^, in which the stochastic elements are overlapped with the finite difference zones. A detail of the procedure is presented step-wise as is shown diagrammatically in Fig. 4. And the sequence includes:

(1) Meshing the finite difference grids, and making sure, each element edge size is smaller than the half of the range, which enables the discretization to depict the spatial variability of a geotechnical parameter.
(2) Obtaining second-order stationary Gaussian field through local regression of the borehole data, which is followed by calculating the statistical characteristics of multivariate, *e.g.*, mean value, variance, ranges and cross-correlations, and taking the residual values of the geotechnical parameter as the conditional data.
(3) Generalizing SGS algorithm into multivariate discretization, thus the residual error values would fluctuate around the stepped-drifts.
(4) Invoking FLAC^3D^ with the pre-assigned stepped-drifts, and setting the additional variations of the residual error.
(5) Stochastically calculating the maximal ground surface settlement induced by shield tunneling process.
(6) Repeating steps 3–5 until the simulation number $N_{tot}$ of MC algorithm is achieved.
(7) Sequentially calculating the failure probability according to the maximal threshold of ground surface settlement.

It is worth noting that if the failure probability equals to $P_{f}=1\times 10^{-k}$, where k is the amplitude of the ten-based exponent, MC algorithm needs to randomly simulate as many as a total of $N_{tot}\approx 1\times 10^{k+2}$ times, otherwise this task becomes quite difficult to be accomplished or practically impossible.

### 3.4 Subset Monte-Carlo simulation

SMC simulation belongs to acceleration sampling algorithm based on Markov chain theory (Papaioannou and Straub, 2012). Systemic failure event F could be denoted by a continuous cascade multiplication of M failure events in a probabilistic space,

(15) $$F=\overset{M}{\underset{i=1}{\text{I}}}F_{i}\text{.}$$

Furthermore, joint probability of a systemic failure is derived,

(16) $$P_{f}\left(\overset{M}{\underset{i=1}{\text{I}}}F_{i})=P_{f}(F_{1}\right)\prod_{i=2}^{M}P_{f}(F_{i}|F_{i-1})\text{.}$$

Thus, the systemic failure could be denoted by G≤0 in Eq. (1). And cascade failure events are defined as $F_{i}=\left\{G\leq g_{i}\right\}$, i=1,...,M, $g_{1}>...>g_{i}>...>g_{M}\leq 0$. Limit state function value $g_{i}$corresponds to the failure ratio $p_{0}$ of subset samples. It is worth mentioning that the initial failure probability $P_{f}\left(F_{1}\right)$ is calculated directly by the MC simulation, and in the steps:

(1) Calculating the probability $P_{f}\left(F_{1}\right)$ of the initial failure events. Taking N stochastic simulation to calculate the limit state function G. $g_{i}$ equals to the $\left\{g_{i}:i=1,...,N\right\}$ of the sequence $\left[\left(1-p_{0}\right)N\right]$, in which case the failure ratio $p_{0}$ and simulation N of the subset algorithm are determined by the trial-and-test procedure.
(2) In the first step of calculating the failure probability, $P_{f}\left(F_{1}\right)$, samples of $i>p_{0}N$ should subject to $G\leq g_{1}$. Moreover, the above $p_{0}N$ samples are considered as the subset seeds (*i.e.*, conditional data) into calculating the consequent failure probability $P_{f}\left(F_{2}\right)$, in which the SGS algorithm would generate other $\left(1-p_{0}\right)N$ realization given the above conditional data.
(3) Repeat the above SMC simulation to iteratively calculate the failure events $F_{3},F_{4},...,F_{M}$ until $g_{M}\leq 0$, and the systemic failure probability approximates to,

(17) $${P_{f}\left(F\right)\approx P_{f}\left(F_{0}\right)}^{M-1}P_{f}\left(F_{M}|F_{M-1}\right)\text{,}$$

where, failure probability $P_{f}\left(F_{M}|F_{M-1}\right)$ equals to the samples of $G_{M}\leq 0$ divided by the subset quantity N at the last step_._ In order to approach the failure probability $P_{f}=1\times 10^{-k}$, classical MC algorithm needs total $N_{tot}=1\times 10^{k+2}$ samples, and when the SMC algorithm is implemented, it improves the effective sample size as shown in Eq. (18),

(18) $$N_{tot}\approx k\left(1-p_{0}\right)N+N\text{.}$$

## 4 Example

**Fig. 5:**
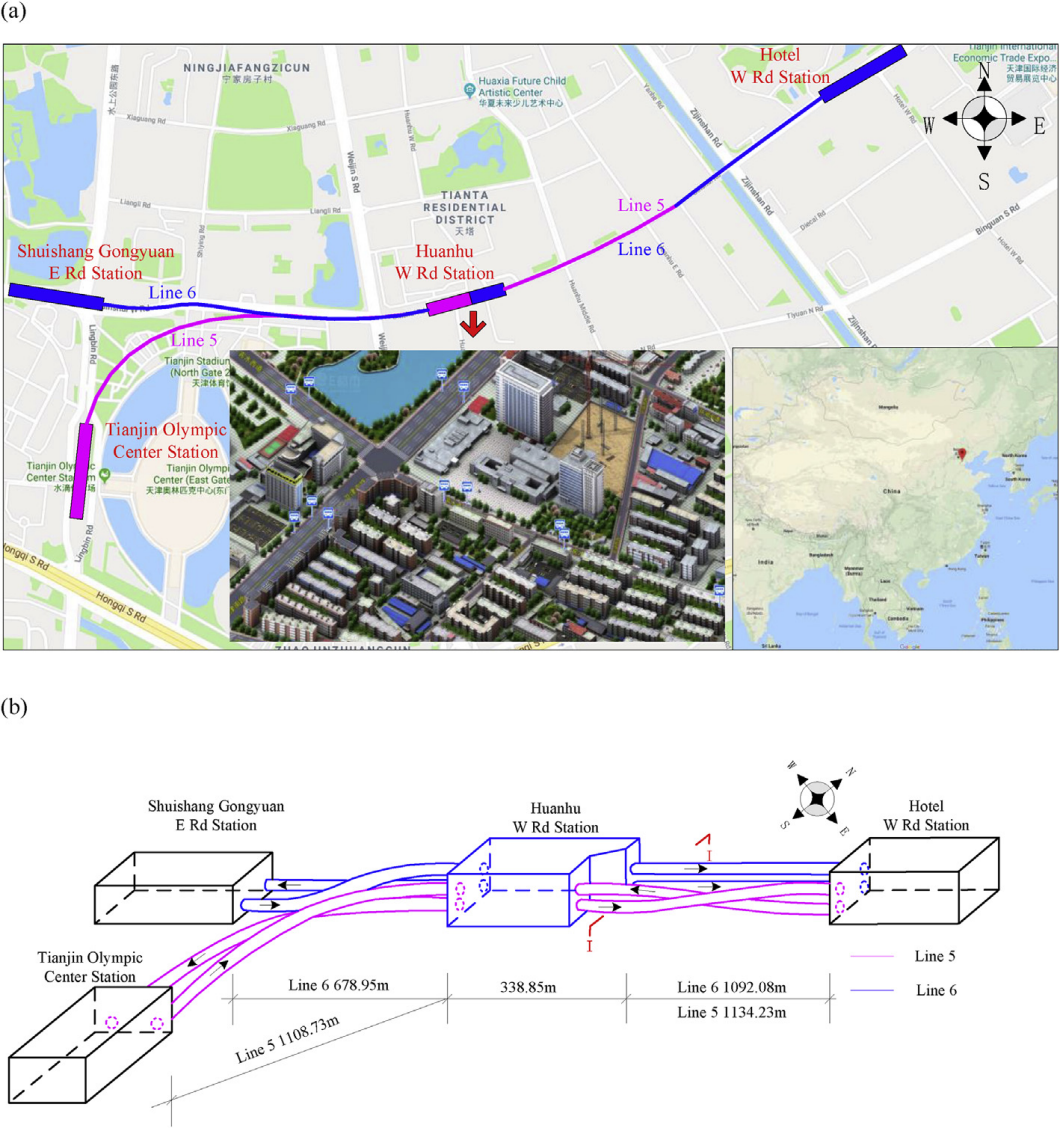
The 5^th^ and 6^th^ metro lines are intersecting to Huanhu W Rd Station in Tianjin, China. (a) Two-dimensional alignments; and (b) Three-dimensional layout.

The 5^th^ and 6^th^ metro lines are intersected at Huanhu W Rd station with four shield tunnels in Tianjin, China (*i.e.*, as shown in Fig. 5a), where the central lines twist and overlap with each other in a narrow underground space that provides convenience for passenger exchange (*i.e.*, as shown in Fig. 5b). At this site, there is a traffic artery and densely packed residential buildings above the subway zone. Hence, quantitative settlement analysis of the ground surface would effectively prevent construction risks. There are two typical interval sections of shield tunneling between Huanhu W Rd station and Binguan W Rd station, which include the overlapping-section I-I and crossing-section II-II. The study concentrates on section I-I due to its larger tunneling disturbance compared with section II-II.

### 4.1 Basic design information

**Fig. 6:**
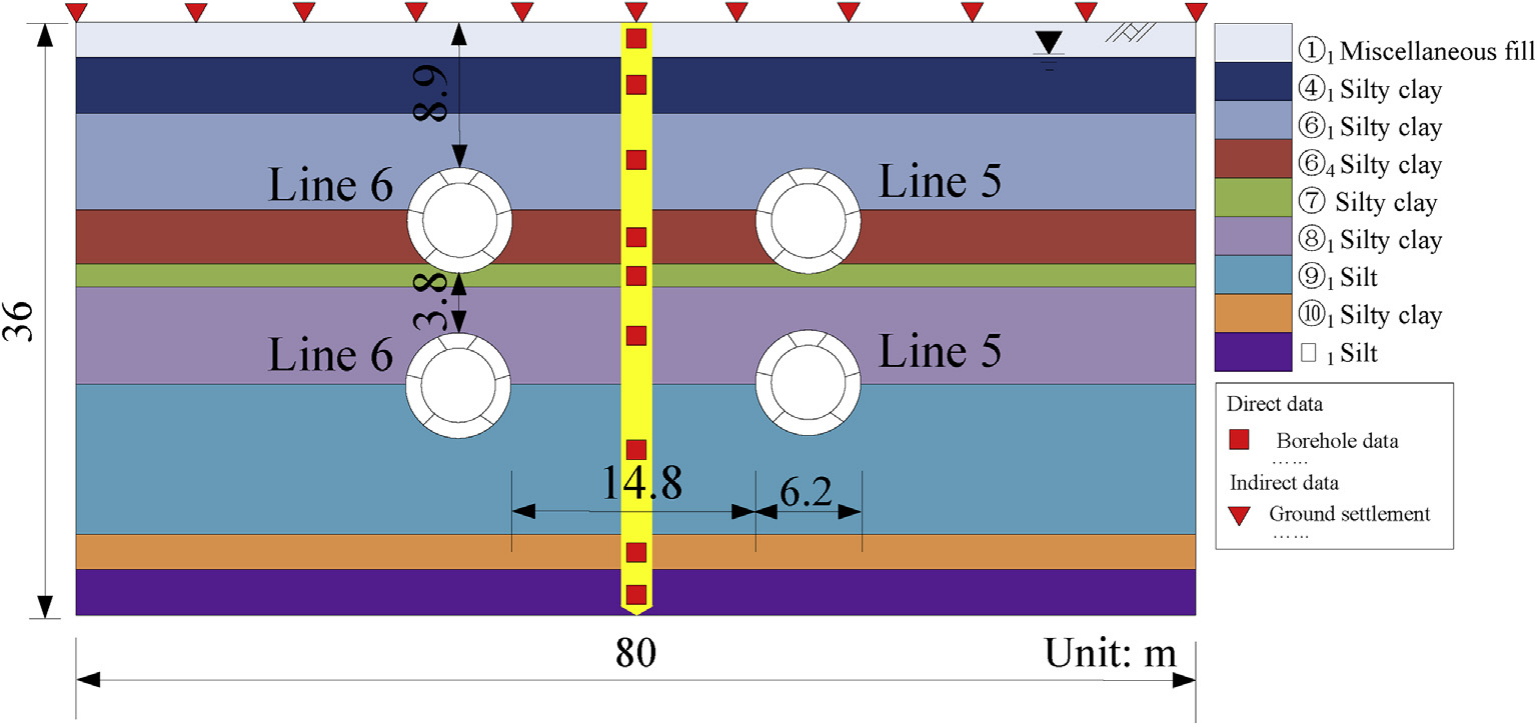
The closest overlapping tunnel section I-I with borehole data.z

Site investigation report provides 84 groups of geotechnical parameters (*e.g*., c,φ and E) from the consolidated-undrained triaxial tests for which the basic soil properties are presented in Table 1. Shield tunnels are designed into 6 reinforced concrete segments as shown in Fig. 6. The inner and outer diameters is adopted by 5.5m and 6.2m, respectively. Table 2 shows the elastic properties of shield machine shell, the concrete segment, and Young's modulus of the segment juncture which is reduced to 70% of the normal value.

**Table 1:** Average values of the basic geotechnical parameters.

Soil layer	H/(m)	ρ/(kg/m^3^)	ν	c/(kPa)	φ/(°)	E/(MPa)
①_1_ Miscellaneous fill	2.2	1800	0.3	4.0	7.0	16.0
④_1_ Silty clay	3.5	1970	0.3	8.0	9.0	30.4
⑥_1_ Silty clay	5.8	1900	0.3	11.0	15.0	28.4
⑥_4_ Silty clay	3.1	1950	0.3	15.0	14.0	29.2
⑦ Silty clay	1.6	1990	0.3	15.0	13.0	26.8
⑧_1_ Silty clay	5.8	1990	0.3	12.0	12.0	44.4
⑨_1_ Silt	9.0	2010	0.3	16.0	28.0	98.4
⑩_1_ Silty clay	2.0	2020	0.3	19.0	17.0	33.2
⑪_1_ Silt	3.0	2000	0.3	16.0	33.0	114.4

**Table 2:** Mechanical parameters of the concrete segment and shield machine shell.

Elastic material	ρ/(kg/m^3^)	Poisson ratio ν	E/(MPa)
Concrete segment	2450	0.17	2.1×10^4^
Shield machine shell	8000	0.22	2.06×10^5^

#### 4.1.1 Numerical analysis schema

Software FLAC^3D^ (Itasca, 2013) is employed to simulate the shield excavation. Numerical excavation moves 2.0m forward at each step, and the tunneling pressure of the bottom and top face is kept at 240kPa and 140kPa, respectively. In this situation, the penetration rate adopts 3 h per ring, and the Grout pressure is maintained at 300kPa of the surrounding 0.2m soil mass. The unfilled gaps in the shield tail are assumed as 20% and are considered to be circularly homogenous void, in which low hydraulic conductivity is assigned.

**Fig. 7:**
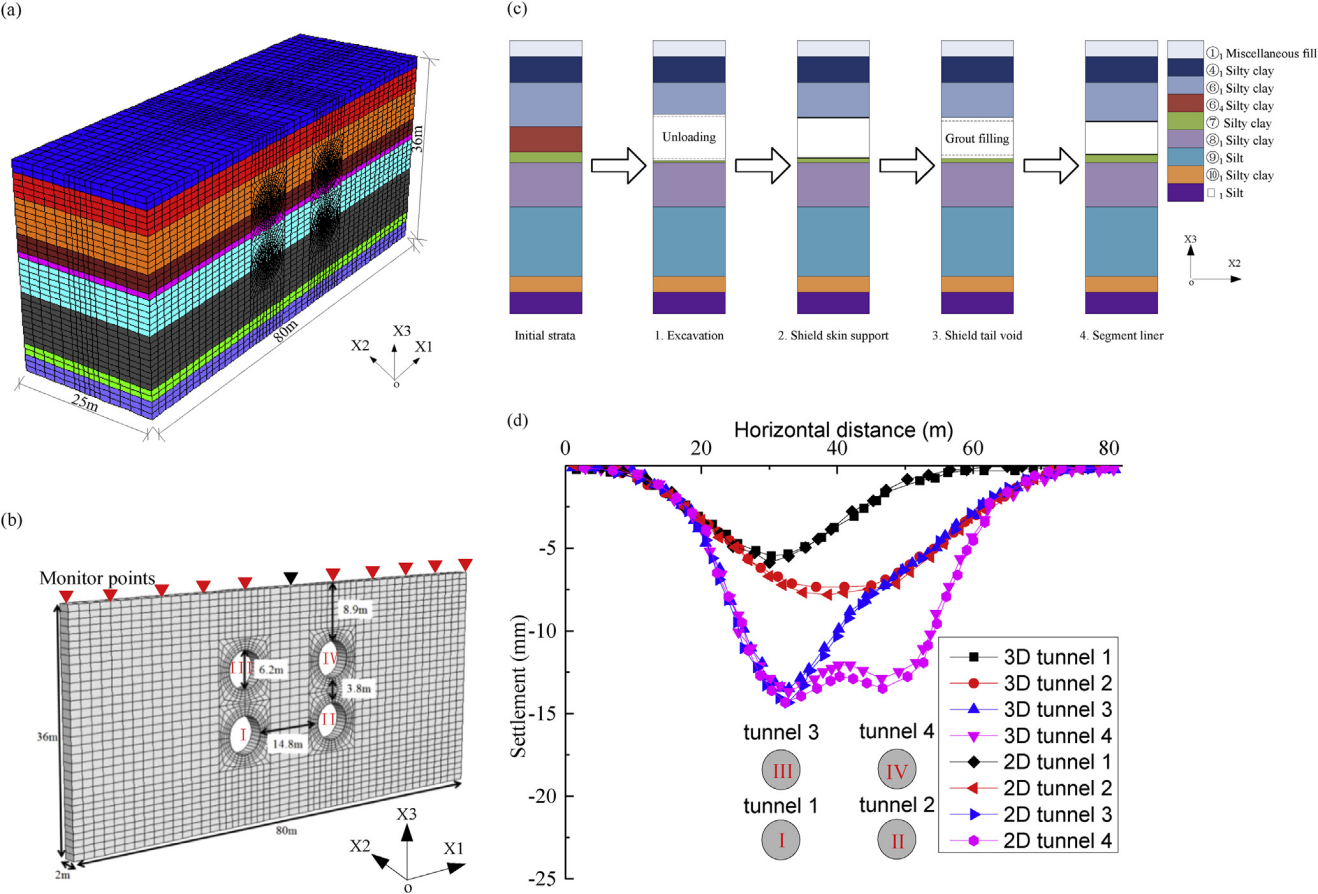
Numerical analysis schema. (a) Three-dimensional model; (b) Two-dimensional model; (c) Shield tunneling process; and (d) Predicted settlement comparison between three-dimensional model and two-dimensional model.

Three-dimensional simulation of soil-structure interaction is shown in Fig. 7(a). The process is very time consuming, four-tunnel excavation analysis of 25m would spend 24 h on a desktop computer with 6 cores i7-4790 CPU at 3.6 GHz. From the time-saving aspect, two-dimensional model is consisted of 5926 nodes and 2880 hexahedral elements as shown in Fig. 7(b), which is only needed 6 min to analyze the closest section I-I. The width and depth adopted for the excavation model is 80m and 36m, respectively; and the meshes around the tunnels are more refined to reduce possible computation errors due to the large displacement gradients in the core areas. Horizontal restraints are set to the left and right, front and back boundaries, and total restraints are set for the bottom boundary.

The tunneling process consisted of four stages as shown in Fig. 7(c): soil removal, shield enclosure, shield tail separation, and segment liner assembly. The Mohr-Coulomb failure criterion is introduced to simulate the soil behavior. Although more complicated models, *e.g*., stiffness hardening soil model (Finno and Calvello, 2005) and models considering the consolidation process have been proposed to simulate the geomechanics under excavation conditions, but more input quantities are required which make the stochastic analysis more time-consuming. Nevertheless, Mohr-Coulomb failure criterion is used commonly for solving many excavation problems (Chowdhury et al., 2013; Orazalin et al., 2015). Furthermore, Juang et al. (2018) reported the uncertainty of the model in geotechnical engineering and indicated that complex model does not always outperform a simple one. As a result, simple geotechnical parameters are often commonly used for Mohr-Coulomb failure criterion, and for this reason they are measured from the current site investigation. Fig. 7(d) indicates that two-dimensional model could subrogate the three-dimensional one to predict the ground surface settlements induced by shield tunneling process, which depends on the equivalent stress–releasing principle. The relative prediction error would be limited into less than 3%.

#### 4.1.2 Sensitivity analysis

**Fig. 8:**
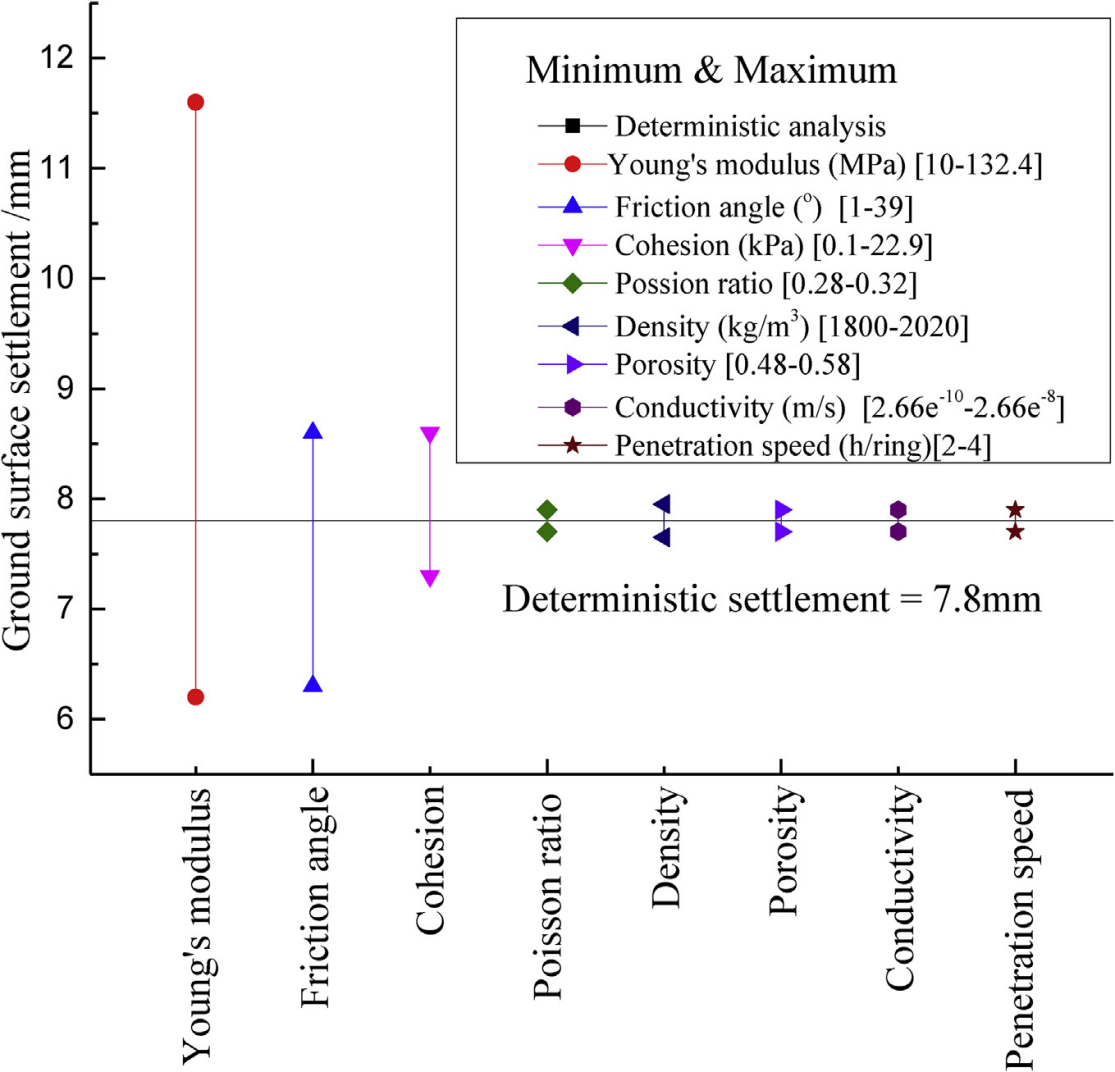
Sensitivity analysis of the key variables to the soil-structure mechanics.

Sensitivity analysis is performed by the three-dimensional model, cohesion c, internal friction angle φ, Young's modulus E, Poisson ratio ν, unit density ρ, porosity n, hydraulic conductivity K and penetration speed s are considered. The calculated deterministic results are depicted in Fig. 8, in which vertical lines represent the minimal and maximal values of the ground surface settlement caused the first shield tunnel. The influence of top-three geotechnical parameters are drawn: Young's modulus E, cohesion c and internal friction angle φ, thus which are denoted as spatial random fields whereas the values of other geotechnical parameters are kept to be constant.

#### 4.1.3 Statistics of the spatial random fields

**Fig. 9:**
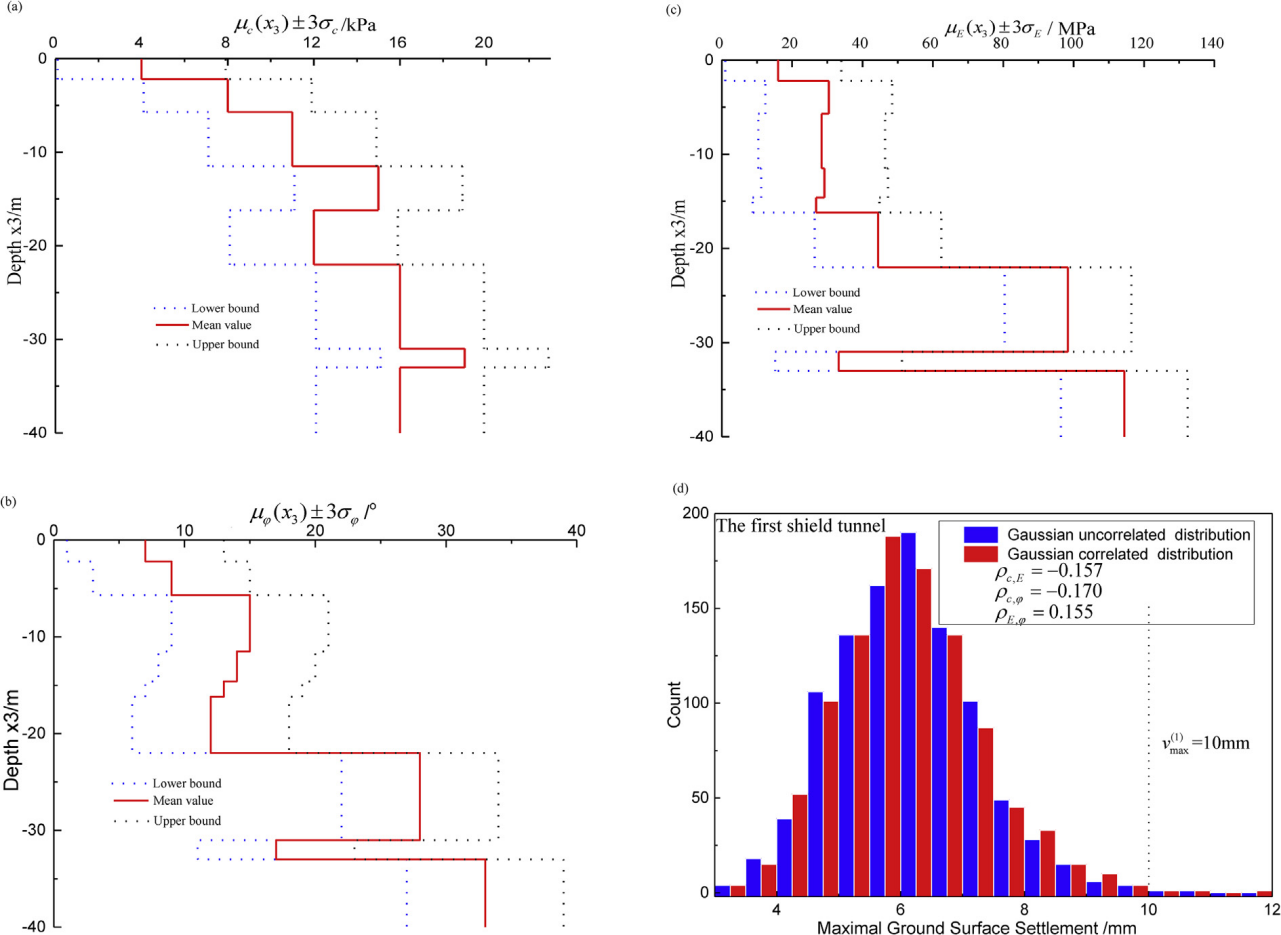
Gaussian characteristics of the key geotechnical parameters. (a) Stepped-mean curves ±3 time standard deviation of cohesion c(kPa); (b) Stepped-mean curves ±3 time standard deviation of internal friction angle φ(^o^); (c) Stepped-mean curves ±3 time standard deviation of Young's modulus E(MPa); and (d) 1000 random predictions of maximal ground surface settlement in the case of the first shield tunneling step, threshold is adopted $v_{\max}^{\left(1\right)}=10\text{mm}$.

section I-I is denoted as the most dangerous overlapping case. Except for the general site investigation, there is one additional borehole to provide conditional data z for the key geotechnical parameters as shown in Table 3. Nine similar soil layers exist in the background project, *i.e*., miscellaneous fill, silty clay, silt, and silty clay from the surface to bottom. Fig. 9(a), (b) and (c) represent the three stepped-drift curves $\mu_{c}\left(x_{3}\right)$, $\mu_{\varphi }\left(x_{3}\right)$ and $\mu_{E}\left(x_{3}\right)$±3 time $\sigma_{c}$, $\sigma_{\varphi }$ and $\sigma_{E}$ along the vertical coordinate X3. Fig. 9(d) shows 1000 random predictions of maximal ground surface settlement according to the classical statistics, and threshold $v_{\max}^{\left(1\right)}=10\text{mm}$. It is obvious that uncorrelated Gaussian distributions of the three key geotechnical parameters would result in a smaller failure probability (*i.e.*, 3% odds of risk) than the real correlations with $\rho_{c,E}=-0.157$, $\rho_{c,\varphi }=-0.170$ and $\rho_{E,\varphi }=0.155$.

**Table 3:** Borehole data of the key geotechnical parameters in section I-I.

Borehole data (Conditional data)	Coordinates			c(kPa)	φ(^o^)	E(MPa)
	*x*_1_(m)	*x*_2_(m)	*x*_3_(m)			
$z_{1}$	40. 000	1. 000	-1. 100	1. 0	6. 4	11. 5
$z_{2}$	40. 000	1. 000	-3. 950	8. 7	5. 9	34. 4
$z_{3}$	40. 000	1. 000	-8. 600	10. 2	16. 9	36. 0
$z_{4}$	40. 000	1. 000	-13. 050	16. 5	14. 4	33. 9
$z_{5}$	40. 000	1. 000	-15. 400	13. 8	12. 0	33. 5
$z_{6}$	40. 000	1. 000	-19. 100	11. 2	8. 8	46. 7
$z_{7}$	40. 000	1. 000	-26. 500	16. 5	28. 3	104. 9
$z_{8}$	40. 000	1. 000	-32. 000	19. 2	16. 5	32. 4
$z_{9}$	40. 000	1. 000	-34. 500	15. 0	35. 1	105. 0

Table 4 lists the statistical characteristics of the spatial random fields, which includes the key geotechnical parameters and model factor. The initial reconnaissance report provided the mean value, variance, maximum and minimum of the nine soil layers, and the statistical characteristics of spatial random fields, *e.g*., vertical range ratios$\left[\eta_{3}\left(c\right),\eta_{3}\left(\varphi \right),\eta_{3}\left(E\right)\right]$, correlation coefficients [ρ(c,φ),ρ(c,E),ρ(φ,E)] and model factor χ come from a Bayesian analysis that assimilated the borehole data and monitoring measurements. Consequently, the classical statistics, spatial random fields would be used to calculate the failure probability of the maximal ground surface settlement.

**Table 4:** Statistical characteristics of the spatial random fields.

Variable	$\mu \left(x_{3}\right)$	$\sigma^{2}$	$a_{1}=a_{2}$(m)	$\eta_{3}$	Correlation coefficient ρ		
					c(kPa)	φ(^o^)	E(MPa)
c/(kPa)	$\mu_{c}\left(x_{3}\right)$	1.3^2^	20	0.404	1	-0.170	-0.157
φ/(^°^)	$\mu_{\varphi }\left(x_{3}\right)$	2.0^2^	20	0.171	-0.170	1	0.155
E/(MPa)	$\mu_{E}\left(x_{3}\right)$	6.0^2^	20	0.390	-0.157	0.155	1
χ	0.749	0.103^2^	-	-	-	-	-

### 4.2 Results and discussion

Based on the local experience of shield tunneling control in Tianjin, China, the upper limit of the first shield tunneling induced ground settlement is adopted the threshold with $v_{\max}^{\left(1\right)}=10\text{mm}$, and it is increased by 5mm for each shield tunneling step until it reaches $v_{\max}^{\left(4\right)}=25\text{mm}$.

#### 4.2.1 Reliability analysis using classical statistics

**Fig. 10:**
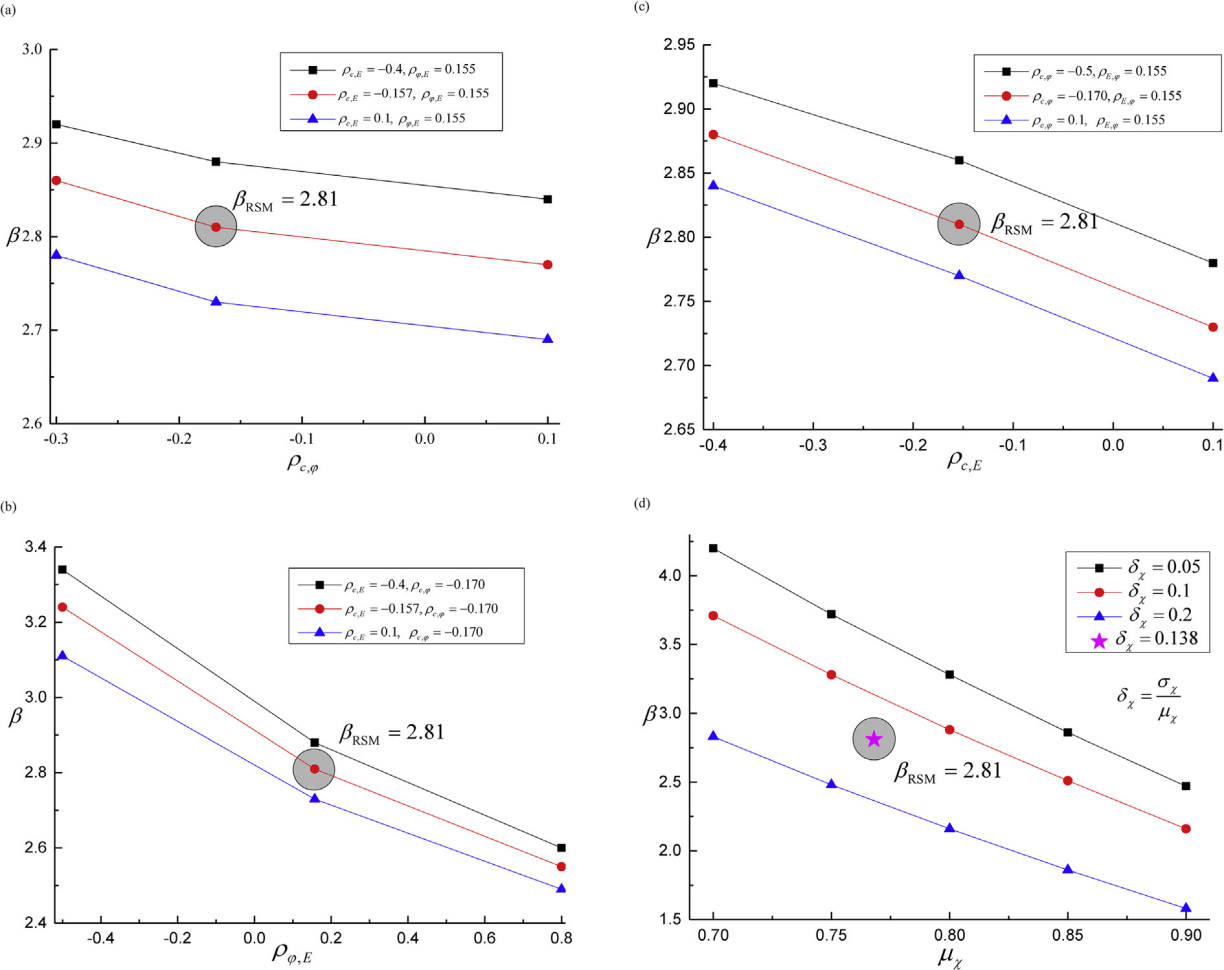
Failure probability of the first shield tunneling step with the key variables (*i.e*., c, φ, E and χ) using RSM of classical statistics. (a) Impacts of $\rho_{c,\varphi }$ between cohesion c(kPa) and internal friction φ(^o^); (b) Impacts of $\rho_{\varphi ,E}$ between internal friction angle φ(^o^) and Young's modulus E(MPa); (c) Impacts of $\rho_{c,E}$ between cohesion c(kPa) and Young's modulus E(MPa); and (d) Impacts of model factor χ.

Mollon et al. (2009) illustrated that the failure probability decreases 3%–10% when the corresponding geotechnical parameters c and φ are considered as negatively correlated variables comparing with uncorrelated assumption. Hence, Fig. 10 shows the failure probability variation of the first shield tunnel, the final result equals to β=2.81 with the stepped-drifts: $\mu_{c}\left(x_{3}\right)$, $\mu_{\varphi }\left(x_{3}\right)$, $\mu_{E}\left(x_{3}\right)$, the SD are $\sigma_{c}$, $\sigma_{\varphi }$, and $\sigma_{E}$, the correlation coefficients are $\rho_{c,\varphi }=-0.170$, $\rho_{c,E}=-0.157$ and $\rho_{\varphi ,E}=0.155$. Fig. 10(a) discussed the failure probability β which changes with the correlation coefficient $\rho_{c,\varphi }$ between c and φ. Let $\rho_{c,\varphi }$ change from -0.5 to 0.1, the value of $\rho_{\varphi ,E}$ varies between -0.5 to 0.8 as shown in Fig. 10(b), and $\rho_{c,E}$ changes between -0.4 to 0.1 as shown in Fig. 10(c). Influences of geotechnical parameters to failure probability, from the smallest to the largest impacts are cohesion c, internal friction angle φ, and Young's modulus E. Fig. 10(d) demonstrates the variability of failure probability corresponding to the model factor χ, which dramatically impacts on the failure probability β. These results reach the minimum requirement of Chinese reliability specification, in which the failure probability β should be at least greater than 2.70.

#### 4.2.2 Reliability analysis using spatial random fields

**Fig. 11:**
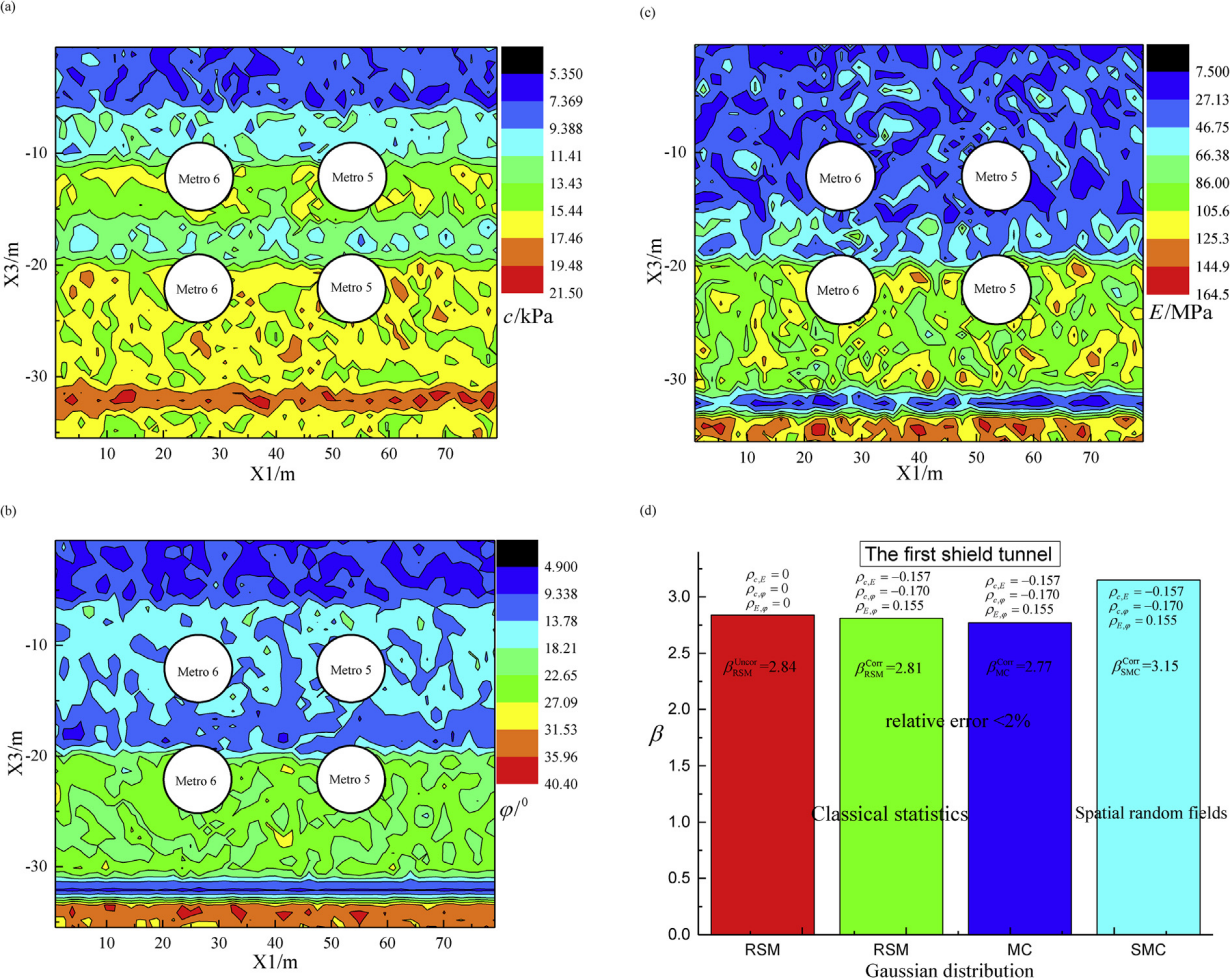
Failure probability of the first shield tunneling step using classical statistics and spatial random fields. (a) Spatially conditional realization of cohesion c(kPa); (b) Spatially conditional realization of internal friction angle φ(^o^); (c) Spatially conditional realization of Young's modulus E(MPa); and (d) Failure probability of the maximal ground surface settlement according to different simulation methods, RSM, MC and SMC algorithms.

Comparison of failure probability for the first shield tunnel in terms of classical statistics and spatial random fields is depicted in Fig. 11. RSM and MC simulations are adopted by classical statistics, whereas SMC simulation is used for spatial random fields. Accordingly, Fig. 11 (a), (b) and (c) present the conditionally spatial realization of c,φ and E, respectively. To validate the effectiveness of RSM, the MC algorithm is taken 100,000 simulations to calculate the failure amplitude at $P_{f}=1\times 10^{-3}$, which has been spending for 11 days and 14 h to finish computation. Furthermore, the differences of failure probability are displayed in Fig. 11(d). There are subtle disparities among $\beta_{\text{RSM}}^{\text{Uncor}}=2.84$, $\beta_{\text{RSM}}^{\text{Corr}}=2.81$ and $\beta_{\text{MC}}^{\text{Corr}}=2.77$ (*i.e*., $P_{f}=$2.5%) in which RSM overestimates 2.0% more than the estimation of MC method. It is important to emphasize that this saves 99% running time. Failure probability $\beta_{\text{SMC}}^{\text{Corr}}=3.15$ is obtained by SMC algorithm of spatial random fields, which is obviously larger than the results of the classical statistics. More importantly, the SMC algorithm took 2,300 stochastic calculations, which is only consumed 2.5% running time comparing with the MC algorithm.

**Fig. 12:**
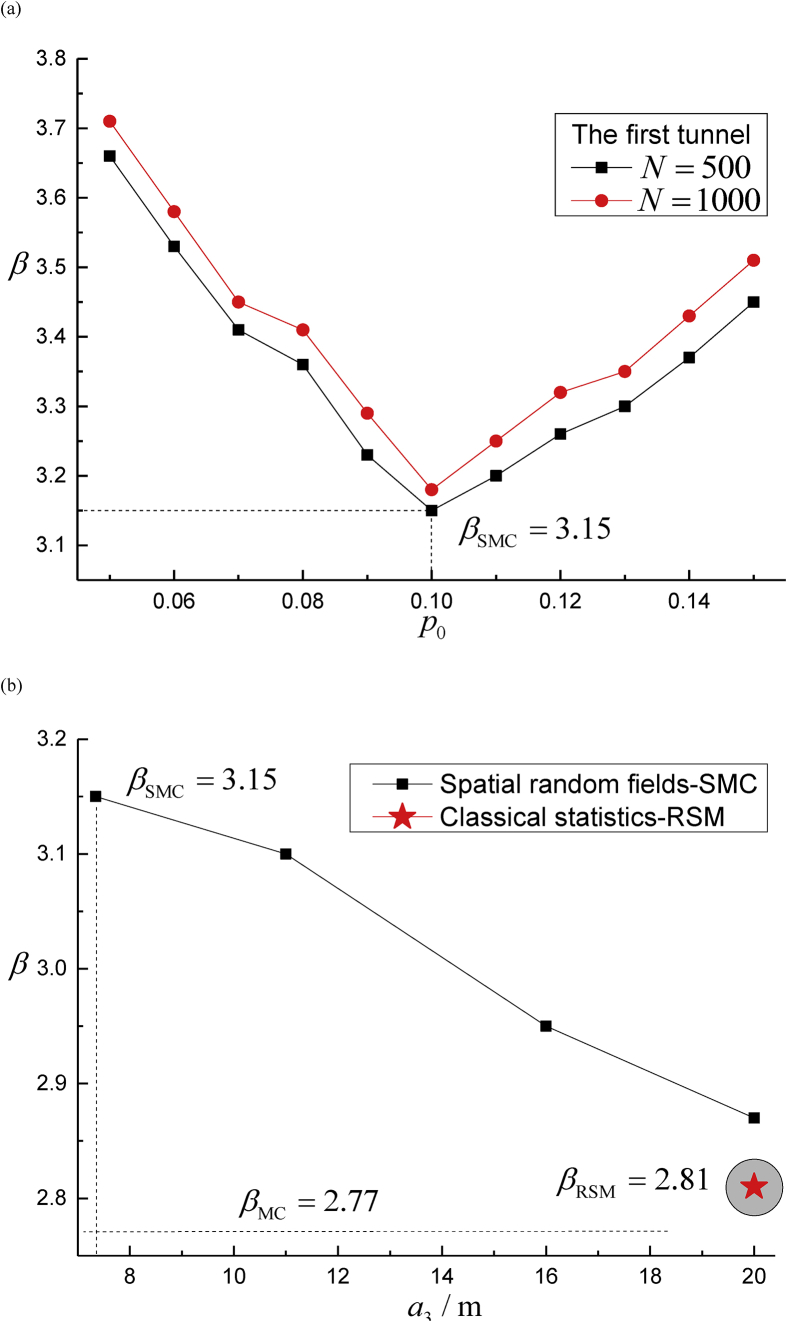
Failure probability of the first shield tunneling step in terms of SMC parameters $p_{0}$, N and vertical range $\alpha_{3}$(m). (a) Impacts of subset parameters $p_{0}$ and N; and (b) Impacts of vertical range $\alpha_{3}$(m) of the key geotechnical parameters.

**Fig. 13:**
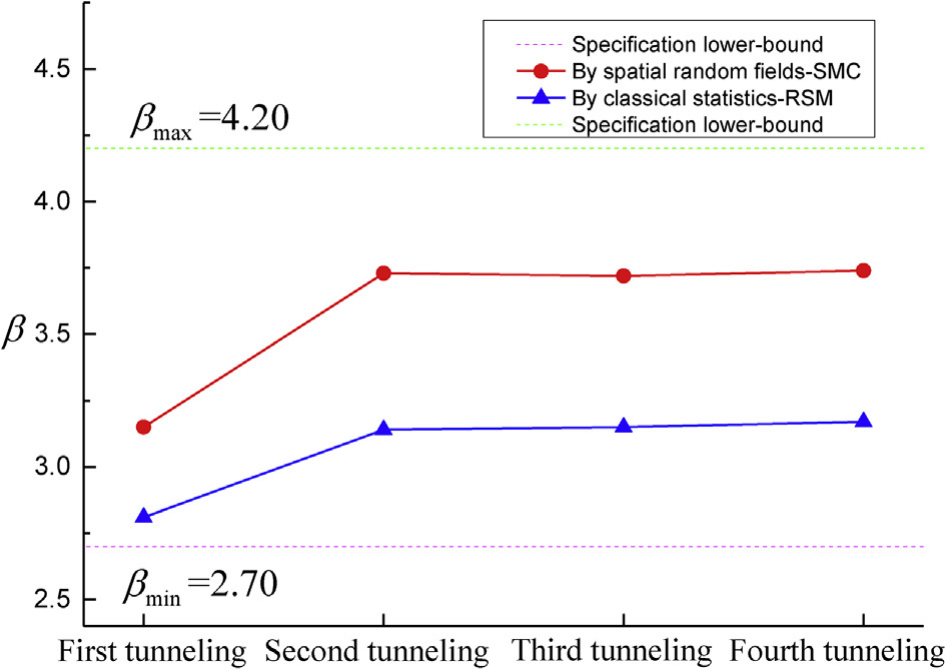
Failure probability of the maximal ground surface settlement is continuously predicted by the key variables (*i.e*., c, φ, E and χ) following the four tunnel excavations.

Two optimal parameters are needed in SMC algorithm: failure ratio $p_{0}$ and subset scale N, and efficiency is presented in Fig. 12(a). Results is affected by Factor $p_{0}$ more than parameter N does. The best parameters are $p_{0}=0.1$ and N=500, and the corresponding result is $\beta_{\text{SMC}}=3.15$. Calculation precision of failure probability from spatial random fields to classical statistics is depicted by Fig. 12(b), the β value decreases dramatically when the range value $\alpha_{3}$ of the spatial random fields increases. This conclusion coincides with the results of classical statistics when range equals to 20m. In other words, random variable is a particular scenario of spatial random field when the range becomes large enough, which proves that more conservative strategy is adopted by the classical statistics whereas the spatial random fields provides more accurate predictions. Finally, the stepwise calculations of the failure probability for both methods are presented in Fig. 13. It should be noted that all of the results reaching the Chinese specification limits between 2.70 to 4.20, and the failure probability converges to a stable value immediately after the second tunnel excavation.

## 5 Conclusions

In the current study, spatial random fields are used to depict the uncertainty propagation of geotechnical parameters, and they are utilized to calculate the failure probability of the 5^th^ and 6^th^ metro lines intersecting at Huanhu W Rd station, Tianjin China. Based on the analysis, the following major conclusions are synthesized:

(1) Surface response method could efficiently calculate the failure probability using first-order second moment, and the result approximates the output of Monte-Carlo Simulation of classical statistics.
(2) Local regression could establish a Gaussian stationary field for multiple soil layers, in which case, variogram and cross-variogram could fully depict the spatial variability of key geotechnical parameters.
(3) Sequential Gaussian simulation could take out multivariate realization of spatial random fields, and subset Monte-Carlo algorithm would efficiently calculate failure probability.
(4) In the application of an overlapping shield tunneling project, the cohesion c, internal friction angle φ, Young's modulus E, and the mechanical model factor χ are determined as unknown variables, In which case, spatial random fields evaluate the failure probability of maximal ground surface settlement between $\beta_{\min}=2.70$ to $\beta_{\max}=4.20$ during the shield tunneling excavations. The final breakthroughs were successfully supported by the results.

Prior knowledge of geotechnical parameters, which plays a critical key role in the early stages of shield tunneling excavation, will be studied in our upcoming research paper.

## Declarations

### Author contribution statement

Baolin Hu: Analyzed and interpreted the data; Wrote the paper.

Changhang Wang: Conceived and designed the experiments; Performed the experiments; Contributed reagents, materials, analysis tools or data.

### Funding statement

This work was supported by the Shanghai Pujiang Program (18PJ1403900), and the Key Laboratory of Geotechnical and Underground Engineering of Ministry of Education, Tongji University (KLE-TJGE-B1706). This work was also supported by the Program for Professor of Special Appointment (Eastern Scholar) at Shanghai Institutions of Higher Learning (2019–26).

### Competing interest statement

The authors declare no conflict of interest.

### Additional information

No additional information is available for this paper.
